# The winding road to platelet α-granules

**DOI:** 10.3389/fcell.2025.1584059

**Published:** 2025-04-16

**Authors:** Andrea L. Ambrosio, Santiago M. Di Pietro

**Affiliations:** Department of Biochemistry and Molecular Biology, Colorado State University, Fort Collins, CO, United States

**Keywords:** platelet α-granule, megakaryocytes, organelle biogenesis, bleeding disorders, NBEAL2, VPS33B/VPS16B, intracellular protein transport

## Abstract

Platelets are anucleate cellular fragments derived from megakaryocytes (MKs) and α-granules constitute their most numerous membrane-bound compartments. These granules play a role in platelet aggregation to form a hemostatic plug but also contain numerous cargo proteins with key functions in angiogenesis, inflammation, wound healing and cancer. Human genetic disorders that cause deficiencies in the biogenesis of platelet α-granules manifest with prolonged bleeding. The initial studies on platelets and MKs from these patients provided a first glimpse into the biosynthesis of α-granules as a membrane trafficking problem. Significant progress in the field has been made in recent years in part due to the creation of iPSC-derived megakaryocytic cells capable of releasing functional platelets, thus overcoming the limitations of working with primary MKs. The emerging model indicates that sorting and recycling endosomes are key intermediate stations traversed by α-granule cargo on their way to the α-granule. Here we describe the different trafficking pathways used by α-granule proteins and elaborate on their commonalities. Similar to other lysosome-related organelles, most of the proteins involved in the biogenesis of α-granules are ubiquitously expressed and we discuss NBEAL2 as a factor highly expressed in MKs that likely diverts this machinery to make α-granules. Importantly, understanding the trafficking pathways involved in the making of the α-granule has an impact not only on platelet biology but may also illuminate the broader lysosome-related organelle field.

## Introduction

Platelets are anucleate cells fundamental for normal hemostasis that contain two types of lysosome-related organelles (LROs): α- and dense (δ-) granules (Blair and Flaumenhaft, 2009). Platelets exert their function in part by releasing the content of these granules in a regulated fashion. (Joshi and Whiteheart, 2017; Jonnalagadda et al., 2012). Each platelet has only 2-8 δ-granules that carry polyphosphate and small molecules such as serotonin, adenosine diphosphate (ADP), adenosine triphosphate (ATP), calcium and zinc (Frojmovic and Milton, 1982; Muller et al., 2009). Alpha-granules on the other hand are more numerous, averaging 40 per platelet, and they contain a variety of protein cargo including not only factors involved in blood clotting (e.g., von Willebrand Factor (vWF) and Factor V (FV)), but also in angiogenesis (e.g., Vascular Endothelial Growth Factor (VEGF), Endostatin, Thrombospondin-1 and Platelet Factor 4 (PF4), inflammation (chemokines such as CXCL1 and Interleukin-8), wound healing (e.g., VEGF and Fibroblast Growth Factor) and cancer (e.g., Platelet-Derived Growth Factor (PDGF), VEGF and RANTES) (Blair and Flaumenhaft, 2009; Frojmovic and Milton, 1982; Pluthero and Kahr, 2023; Sharda and Flaumenhaft, 2018).

Platelets derive from megakaryocytes (MKs), which primarily reside in the bone marrow, and it is in these cells that both α- and δ-granules are synthesized and packaged into proplatelets that are subsequently released into circulation (Patel et al., 2005; Italiano et al., 1999; Hartwig and Italiano, 2003). Primary MKs are not amenable for either cell biology or biochemical analyses, which has been a major obstacle in the study of platelet granule biogenesis. In 2014 however, the creation of an iPSC-derived megakaryocytic cell line (imMKCL) that recapitulates the biogenesis of platelet granules provided a new tool to the field (Nakamura et al., 2014; Ito et al., 2018). Since then, new players and pathways have been discovered and the sorting and recycling endosomes in MKs have been established as key hubs of the α-granule biosynthesis machine (Ambrosio et al., 2022; Ambrosio and Di Pietro, 2019; Lo et al., 2020).

Platelet α-granules are round or ellipsoidal, 200–500 nm in diameter, their lumen is acidic, with an average pH of 5.2, and they contain a heterogeneous population of protein cargo (Italiano et al., 2008; Sehgal and Storrie, 2007; Yao and Kahr, 2025; Pokrovskaya et al., 2020). Both luminal and transmembrane α-granule proteins are either synthesized by the MK, such as P-Selectin, PF4 and vWF, or endocytosed like Fibrinogen, FV, immunoglobulins and albumin (Blair and Flaumenhaft, 2009; Cramer et al., 1989). An endocytic pathway to α-granules is also present in platelets (Behnke, 1992), demonstrated by the increase in accumulation of some endocytosed α-granule proteins as platelets age (Heilmann et al., 1994).

Here, we elaborate on the established knowledge of the intracellular trafficking pathways and machinery used by α-granule proteins to reach the α-granule. We describe mutations in membrane trafficking proteins that underlie platelet α-granule biogenesis phenotypes associated with human genetic disorders manifesting with prolonged bleeding. We also discuss how the ubiquitous sorting endosomal retrieval and recycling machinery is repurposed in MKs to synthesize α-granules, an emergent feature for cells specialized in the biogenesis of LROs. And finally, we comment on how the field is utilizing the knowledge gained about α-granules and their biogenesis to start engineering designer platelets with potential clinical uses.

## Transport to α-granules of plasma proteins taken up by megakaryocytes

The first evidence that indicated α-granules originate from the endolysosomal system came from electron microscopy images following the path and kinetics of gold-labeled bovine serum albumin (BSA) taken up by cultured primary MKs (Heijnen et al., 1998). The authors visualized BSA first populating endosomes, followed by multi-vesicular bodies (MVBs) and finally reaching α-granules. Interestingly, they noticed BSA first in MVBs with typical morphology (MVBI) and then a special type of MVB that contained not only internal vesicles but also electron-dense material (MVBII), which is considered an intermediate maturation compartment between MVBI and the α-granule. Like albumin, immunoglobulin G and other luminal α-granule proteins reflect the composition and concentration of plasma, indicating they are likely acquired by fluid-phase endocytosis (George, 1990; Handagama and Bainton, 1989; Handagama et al., 1989).

Fibrinogen is endocytosed, both in MKs and platelets, bound to its receptor integrin αIIbβ3 with evidence for this process to be both clathrin dependent or independent (Behnke, 1992; Handagama et al., 1993; Gao et al., 2018). The intracellular traffic of αIIbβ3 is regulated by Arf6 and, consistently, the uptake and storage of Fibrinogen is decreased ∼50% in platelets from Arf6 deficient mice (Huang et al., 2016). Additionally, αIIbβ3 recycles from endosomes back to the plasma membrane. In particular, integrin β3 contains a sorting signal in its cytosolic domain that is bound by the endosomal adaptor SNX17 and this suggests that, similar to other integrins, αIIbβ3 is a cargo of the Commander endosomal retrieval and recycling pathway (see “The α-granule biogenesis machinery” section for details) (Tseng et al., 2014; Ghai et al., 2013; Butkovic et al., 2024; McNally et al., 2017).

Another well studied endocytosed protein present in α-granules is FV. Unlike Fibrinogen, FV cannot be endocytosed by platelets but only by megakaryocytes during a specific stage of their differentiation (Bouchard et al., 2005). The endocytosis of FV from plasma is clathrin- and Ca^2+^-dependent and involves LRP-1 and an unidentified, specific FV receptor (Bouchard et al., 2008). It has been shown Galectin-8 also mediates the uptake of FV. Galectin-8 binds FV and it has been proposed it transfers FV to LRP-1 or cross-links FV to integrins (Zappelli et al., 2012). Consistently, Galectin-8 has been shown to bind both LRP-1 and αIIbβ3 and exert its function by protein-sugar and protein-protein interactions (Zick, 2022; Levy et al., 2006). Remarkably, LRP-1 also contains a SNX17 sorting signal in its cytosolic domain and it has been shown to be a cargo of the Commander pathway in other cell types (Butkovic et al., 2024).

## Transport to α-granules of proteins synthesized by megakaryocytes

Alpha-granule proteins, either soluble or transmembrane, traverse the endoplasmic reticulum (ER) and Golgi apparatus where they are post-translationally modified, however their topology, size and individual characteristics are unlikely satisfied by a single route to the α-granule (Blair and Flaumenhaft, 2009; Yao and Kahr, 2025). Also, there is no known universal sorting signal associated with α-granule proteins that would mediate transport to α-granules. Immunoelectron microscopy images of Golgi-associated vesicles containing α-granule proteins led to the conclusion that α-granule cargo was delivered from the Golgi directly to MVBs (Blair and Flaumenhaft, 2009; Cramer et al., 1989). However, live cell fluorescence microscopy analysis of imMKCL cells showed that newly synthesized α-granule proteins traffic through endosomes before reaching MVBs (Ambrosio et al., 2022; Ambrosio and Di Pietro, 2019). In particular, after leaving the Golgi, the luminal α-granule protein PF4 traverses the Rab5 sorting endosome followed by the Rab11 recycling endosome before reaching a Rab7 MVB and finally arriving at α-granules (Ambrosio and Di Pietro, 2019).

Despite the recent advances described above, the traffic of luminal, MK-made proteins to the α-granule is not completely understood. Nevertheless, evidence obtained from several groups studying various cargoes suggests these proteins must be actively retained by MKs (Chanzu et al., 2021; Lykins et al., 2025; Lo et al., 2018). However, how these luminal cargoes are linked to the cytosolic α-granule biogenesis machinery remains unknown. The current consensus idea is that α-granule luminal proteins either concentrate on electrostatic “sponges” or self-aggregate to reach the α-granule (Chanzu et al., 2021; Lykins et al., 2025; Cramer et al., 1985; Blagoveshchenskaya et al., 2002; Wagner et al., 1991). The proteoglycan serglycin (SG) is an example of an electrostatic “sponge”. Binding to SG is required for the packaging of small positively charged chemokines into α-granules which is evident by the reduction of PF4, PDGF, and β-Thromboglobulin in SG deficient mouse platelets (Chanzu et al., 2021; Lykins et al., 2025; Woulfe et al., 2008). Also, Thrombospondin-1, that contains a positively charged heparin-binding domain, has been shown to highly colocalize with SG by structured illumination microscopy of human platelets (Pluthero and Kahr, 2023). SG is a small polypeptide (18 kDa) produced in both hematopoietic and non-hematopoietic cells and decorated with tissue-specific glycosaminoglycan chains with different sulfation patterns (Kolset and Gallagher, 1990; Schick et al., 2001; Kolset and Pejler, 2011). It can accommodate several PF4 molecules that interact electrostatically with its sulfated glycans chains (Lord et al., 2017).

Supporting the importance of the electrostatic “sponge” mechanism, it has been shown SG deficient MK are unable to retain PF4, which is then secreted to the interstitial fluid of the bone marrow altering bone marrow morphology and affecting the homeostasis of the hematopoietic stem cell niche (Chanzu et al., 2021; Lykins et al., 2025). Importantly, although at significantly lower levels than SG deficient MK, wild type MK also release PF4 in the bone marrow (Chanzu et al., 2021; Bruns et al., 2014; Lambert et al., 2007). Intramedullary PF4 has a negative paracrine effect on megakaryopoiesis and it is endocytosed and stored in α-granules in, at least in part, an LRP-1-dependent pathway (Bruns et al., 2014; Lambert et al., 2009; Lambert et al., 2015). Additionally, a hydrophilic loop present in PF4 and other chemokines has been proposed to target these molecules to the α-granule but no receptor or mechanism has been elucidated (El Golli et al., 2005).

An example of self-aggregation as a proposed mechanism aiding α-granule targeting comes from vWF. This large, multimeric glycoprotein is found in eccentric internal structures within α-granules that resemble tubules formed by vWF in another LRO: the Weibel-Palade body (WPB) in endothelial cells (Cramer et al., 1985; Huang et al., 2008). The heterologous expression of vWF can induce the formation of storage organelles in cells that contain a regulated secretory pathway, potentially through an aggregation event that involves its propolypeptide (Blagoveshchenskaya et al., 2002; Wagner et al., 1991). Although, the existence of a putative targeting sequence within its propolypeptide has not been discarded (Wagner et al., 1991). Multimerin, a FV binding protein, is another large glycoprotein proposed to be sorted by homoaggregation (Hayward et al., 1999). Similar to vWF, in resting platelets Multimerin accumulates in the peripheral, electron-lucent zone of α-granules complexed to FV (Hayward et al., 1995).

Several of the transmembrane proteins present on the α-granule limiting membrane are also expressed on the plasma membrane of both MK and platelets (Berger et al., 1996). This is the case of integrins such as αIIbβ3, α2β1 or α6β1, the Thrombospondin-1 receptor CD36, the tetraspanin CD9, the adhesion molecule PECAM1, and GPIb-IX-V, the receptor for vWF (Berger et al., 1996; Cramer et al., 1990; Maynard et al., 2010; Berger et al., 1993; Cramer et al., 1994). In some instances, these proteins are known to be the endocytic receptors for luminal α-granule components, e.g., αIIbβ3 is the receptor for extracellular Fibrinogen (Handagama et al., 1993), and it has been speculated they use the same endocytic pathway as plasma proteins to reach the α-granule (Berger et al., 1996). Interestingly, many of these plasma membrane proteins are known integrin β3 and β1 interactors (Miao et al., 2001; Berditchevski, 2001; Jackson, 2003), which suggests they coexist on the same plasma membrane domains and they probably use the same endocytic carriers. On the other hand, other transmembrane proteins like CD40 ligand (CD40L) and P-Selectin, which are also known to bind integrins (Hassan et al., 2022; Andre et al., 2002a; Takada et al., 2023), are stored in α-granules and relocate to the plasma membrane upon platelet activation and granule fusion with the plasma membrane (Andre et al., 2002b).

The transport of P-Selectin to both the α-granule and WPB in endothelial cells, has been studied by several groups (Ambrosio et al., 2022; Lo et al., 2020; Harrison-Lavoie et al., 2006; Hartwell et al., 1998; Modderman et al., 1998). P-Selectin is a single pass transmembrane protein that contains several sorting signals in its cytosolic domain, including signals that mediate endocytosis and binding to the endosomal adaptor SNX17 (Ghai et al., 2013; Modderman et al., 1998; Blagoveshchenskaya et al., 1999). In MKs, P-Selectin recycles from endosomes to the plasma membrane before being stored in α-granules in a process that depends on its cytosolic domain and the Commander retrieval and recycling pathway (Ambrosio et al., 2022). Interestingly, in endothelial cells, a pathway from the plasma membrane to WPB has also been described (Harrison-Lavoie et al., 2006). However, the complete removal of P-Selectin cytosolic domain does not impede its localization to α-granules, demonstrated by experiments using platelets of transgenic mice expressing a truncated version of P-Selecting missing its cytosolic domain. When activated, these platelets expose the same amount of plasma membrane P-Selectin and show a similar level of platelet-leukocyte interaction as wild-type platelets (Hartwell et al., 1998). Similarly, a P-Selectin molecule missing its cytosolic domain is able to reach WPB in human umbilical vein endothelial cells (HUVEC) (Harrison-Lavoie et al., 2006). These pieces of evidence indicate the P-Selectin extracellular domain (equivalent to the α-granule lumen) contains determinants for α-granule/WPB localization.

## The α-granule biogenesis machinery

Similar to other LROs like melanosomes or δ-granules, most of the known proteins involved in making α-granules are ubiquitously expressed and involved in essential membrane trafficking pathways (Sharda and Flaumenhaft, 2018; Bultema et al., 2012; Ambrosio and Di Pietro, 2017; Ambrosio et al., 2012). The discovery that mutations in VPS33B or VPS16B (VIPAS39, VIPAR, SPE-39) are responsible for arthrogryposis, renal dysfunction, and cholestasis (ARC) syndrome provided the first evidence that the biogenesis of α-granules depends on membrane trafficking machinery (Urban et al., 2012; Lo et al., 2005; Gissen et al., 2004). Patients with this autosomal recessive multisystem disorder present with abnormal bleeding due to α-granule deficiency among other severe clinical symptoms (Yao and Kahr, 2025).

VPS33B belongs to Sec1/Munc18 (SM) protein family that regulate soluble N-ethylmaleimide–sensitive factor attachment protein receptor (SNARE) function (Lo et al., 2005). Members of the SM family contribute to the efficiency and accuracy of membrane fusion events mediated by SNARE proteins by acting as templates of these reactions (Baker and Hughson, 2016). VPS33B and VPS16B form a stable complex that does not contain other proteins, in contrast to paralogs VPS33A and VPS16A, which associate with each other and additional proteins into tethering complexes known as HOPS and CORVET (Ambrosio and Di Pietro, 2019; Liu et al., 2023; Hunter et al., 2018; Shvarev et al., 2022). However, VPS33B and VPS16B have been proposed to associate with a 2:3 stoichiometry that would allow simultaneous interaction with two SNARE bundles or SNAREpins (Liu et al., 2023).

VPS33B deficiency in imMKCL cells results in degradation of α-granule proteins in lysosomes, demonstrating the VPS16B-VPS33B complex role is to ensure the accuracy of α-granule protein transport (Ambrosio and Di Pietro, 2019). VPS33B localizes to sorting endosomes where it binds the SNARE protein Syntaxin 12 (Stx12) and the coiled-coil protein CCDC22, one of the components of the Commander complex (Ambrosio and Di Pietro, 2019). The 16 subunit Commander complex is composed of the Retriever sub-complex (VPS26C, VPS35L and VPS29), the dodecameric COMMD/CCDC22/CCDC93 (CCC) sub-complex, and DENND10 (Butkovic et al., 2024). In non-specialized cell types, Commander engages SNX17 and its bound transmembrane protein cargoes, rescuing them from a late endosome/lysosome degradative pathway and instead recycling these proteins to the plasma membrane (Butkovic et al., 2024; McNally et al., 2017). This is also true in MKs but there may be an additional MK-specific Commander function mediating transport to MVBII/α-granules as seen for another LRO: the melanosome (Bajpai et al., 2023). The FERM domain of SNX17 recognizes transmembrane proteins that contain a ØxNxx[Y/F] sorting signal in their cytosolic domain (where Ø is a hydrophobic residue and x is any residue) such as integrins β1 and β3, P-Selectin and LRP-1 (Ghai et al., 2013). Accordingly, a dominant negative version of SNX17 causes P-Selectin retention in endosomes (Ambrosio et al., 2022). VPS33B interactors Stx12 and COMMD3, a member of the CCC complex, are needed for normal α-granule biogenesis. Also important, Stx12 and the CCDC22 subunit of CCC compete for binding to VPS33B, suggesting a hand-off mechanism in which the fusion of vesicles containing α-granule cargo with endosomal compartments is coupled with the retrieval machinery thus ensuring cargo proteins escape lysosomal degradation (Ambrosio et al., 2022). The WASH complex is required for endosomal deposition of F-actin and cargo trafficking and is a crucial component of the Commander pathway (Simonetti and Cullen, 2019). In agreement, it was recently reported that MK- and platelet-specific WASH deficient mice have a selective reduction of αIIbβ3 expression with a concomitant αIIbβ3 mislocalization to internal membranes and a delay in Fibrinogen uptake (Schurr et al., 2023).

NBEAL2 is the only other known protein that when mutated causes α-granule biogenesis defects both in mice and humans. Mutations in NBEAL2 were identified in Gray Platelet Syndrome (GPS) patients, whose platelets appear gray on electron microscopy images due to the absence of α-granules (Albers et al., 2011; Gunay-Aygun et al., 2011; Kahr et al., 2011). In contrast with VPS33B and VPS16B, NBEAL2 is mainly expressed in hematopoietic cells and therefore GPS symptoms affect the hematopoietic system and include thrombocytopenia, excessive bleeding and myelofibrosis (Yao and Kahr, 2025).

NBEAL2 is a multidomain, large, cytosolic protein and belongs to a group of 9 Beige and Chediak-Higashi syndrome (BEACH) domain containing proteins (Pereira and Gershlick, 2024). Members of this group have been associated with processes that involve membrane trafficking such as regulation of vesicles fusion and fission, autophagy and antigen cross-presentation (Cullinane et al., 2013; Simonsen et al., 2004; Theisen et al., 2018). Alphafold modelling of these proteins predicts they share an alpha-solenoid/beta-propeller molecular structure which indicates they may have a protocoatomer origin and function as membrane coat proteins (Pankiv et al., 2024). Generally, coat proteins are master membrane trafficking regulators involved in cargo selection and concentration, membrane deformation, differentiation of subdomains within organelles and association of carriers with both the cytoskeleton and the acceptor organelle (Bonifacino and Lippincott-Schwartz, 2003). Consistently, pulldown experiments show NBEAL2 binds P-Selectin using both human platelets extracts and over-expressed recombinant proteins in non-specialized cells (Lo et al., 2020; Lo et al., 2018). Also, NBEAL2 has been reported to associate with the ER protein SEC22B and deletion of this ER protein from imMKCL cells results in failure of α-granule formation (Lo et al., 2020). Additionally, a proximity ligation screening identified Dock7, Sec16a, and Vac14 as NBEAL2 interactors (Mayer et al., 2018). Immunofluorescence microscopy of primary MKs show NBEAL2 colocalizes with P-Selectin and SEC22B, pointing towards a role of ER contact sites in α-granule biogenesis (Lo et al., 2020). HeLa cells stably expressing recombinant NBEAL2 show it associates predominantly with Rab11 and Rab38 compartments, suggesting endosomal localization (Pankiv et al., 2024).

NBEAL2 deficient mice MKs do not retain soluble α-granule cargo, either endocytosed (Fibrinogen) or MK-made (PF4) (Lykins et al., 2025; Lo et al., 2018). The release by MK of α-granule proteins, such as growth factors and cytokines, into the bone marrow explains the myelofibrosis presented in GPS patients (Yao and Kahr, 2025). P-Selectin on the other hand, is not completely depleted from NBEAL2 deficient mouse platelets, indicating NBEAL2 functions downstream VPS16B/VPS33B in the α-granule biogenesis pathway (Kahr et al., 2013).

## Current model for the transport of α-granule proteins in megakaryocytes

**FIGURE 1:**
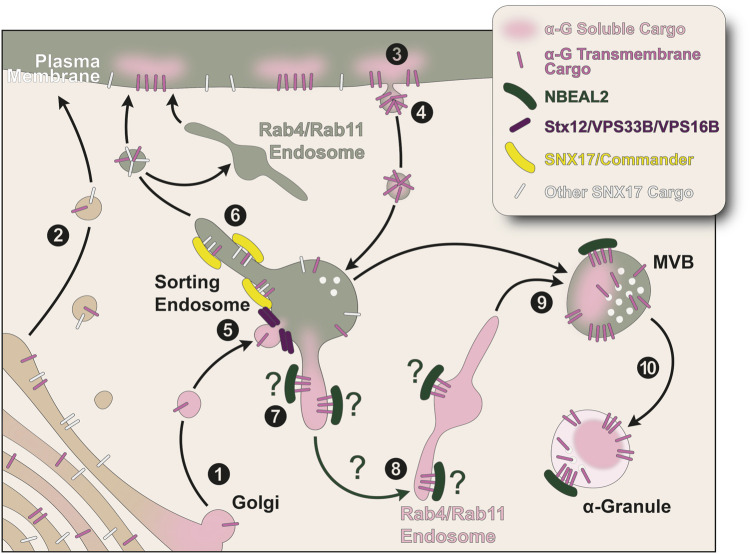
Model for transport of α-granule proteins in megakaryocytes. All MK-synthesized α-granule proteins traverse the ER and the Golgi. (1) Presumably, luminal α-granule proteins homoaggregate and/or concentrate in subdomains of the Golgi apparatus where they are packaged in carriers destined to sorting endosomes. Transmembrane α-granule proteins may also be included in these vesicles. Alternatively, (2) transmembrane proteins enter a default secretory pathway to the plasma membrane and, like luminal α-granule cargo, (3) reach the sorting endosome by endocytosis. (4) We speculate α-granule transmembrane proteins containing similar sorting signals are incorporated in plasma membrane patches and endocytosed in the same transport carriers. (5) Normal fusion of vesicles containing α-granule proteins with endosomes requires the SNARE protein Stx12 and the VPS33B-VPS16B complex. The sorting endosome is the main hub for the transport of α-granule proteins. (6) The endosomal retrieval and recycling Commander complex is responsible for both corralling α-granule cargo away from a degradative fate in lysosomes and the recycling of transmembrane proteins containing a sorting signal in their cytosolic domain that is recognized by SNX17. Sorting endosomes are also the precursor organelle of lysosomes and δ-granules (not depicted), therefore α-granule cargo must be segregated in a process potentially mediated by NBEAL2. (7, 8) We hypothesize NBEAL2 facilitates cargo transfer by binding the cytosolic domain of transmembrane proteins to generate cargo protein carriers destined for α-granules. These tubules and vesicles are Rab4/Rab11-labeled compartments, recycling endosomes that have been repurposed in these cells presumably to enrich and refine the proteins that reach the α-granule. (9) α-granule proteins reach MVBs providing the content for MVBI, containing vesicles but lacking electron dense luminal material, to mature into MVBII, containing both vesicles and electron dense luminal material. The MVBI-MVBII transition is not depicted for simplicity. (10) The maturation of MVBII is the final step in the biogenesis of α-granules. α-G: α-granule; MVB: multi-vesicular body.

Proteins stored in α-granules are made by the MK or by other cells, are soluble or transmembrane, range in size from very large to very small, and perform many different functions in several different pathways (Blair and Flaumenhaft, 2009). However, they all converge in α-granules, which suggests they contain common trafficking labels that target them to this organelle. Most of the known trafficking machinery involved in the α-granule biogenesis pathways are proteins ubiquitously expressed (Ambrosio et al., 2022; Ambrosio and Di Pietro, 2019). Not only that, in MKs these proteins both perform essential cargo sorting functions and make α-granules, pointing towards the existence of a MK-specific factor that coordinates these two processes. NBEAL2 is well positioned to perform this task. It is highly expressed in hematopoietic cells and RNAseq of differentiated imMKCL cells shows its upregulation compared to undifferentiated cells (Ambrosio et al., 2022; Yao and Kahr, 2025). Consistently, GPS patients present with mostly hematopoietic phenotypes (Yao and Kahr, 2025). It has been recently proposed that NBEAL2 functions as an endosomal membrane coat protein (Pereira and Gershlick, 2024; Pankiv et al., 2024). Several reports support this idea. First, in agreement with the cargo selection and concentration function of coat proteins, NBEAL2 binds P-Selectin (Lo et al., 2020; Lo et al., 2018). Second, NBEAL2 deficient MKs secrete luminal α-granule cargo (Lykins et al., 2025; Lo et al., 2018). We speculate NBEAL2 may specify endosomal subdomains where α-granule cargo is both retrieved to avoid secretion and directed towards α-granules (Figure 1).

The high volume of MK-synthesized α-granule cargo in itself is probably a driving force for the creation of α-granules. The luminal aggregation of very large multimeric proteins like vWF or Multimerin and the protein clusters generated by the electrostatic “sponge” SG could aid in the formation of specialized endosomal subdomain (Chanzu et al., 2021; Lykins et al., 2025; Blagoveshchenskaya et al., 2002; Wagner et al., 1991; Hayward et al., 1999; Hayward et al., 1995). But how do these luminal, high-protein concentration domains communicate with the trafficking machinery on the cytosolic side of the membrane? A common denominator across all different α-granule proteins’ types is their direct or indirect association with integrins (Miao et al., 2001; Berditchevski, 2001; Jackson, 2003; Hassan et al., 2022; Andre et al., 2002a; Takada et al., 2023). Several integrins are present on the α-granule limiting membrane and their luminal/extracellular domains interact with a host of other α-granule cargo, both soluble and transmembrane (Cramer et al., 1990; Maynard et al., 2010). Interestingly, integrins β1 and β3 share some of the same sorting signals in their cytosolic domains as P-Selectin and LRP-1: a receptor involved in the trafficking of FV and PF4 known to interact with integrins (Ghai et al., 2013; Hu et al., 2007; Rabiej et al., 2016). In agreement, a recent report indicates the luminal domain of P-Selectin binds integrins (Takada et al., 2023). An integrin-piggyback mechanism could explain the fact that the cytosolic domain of P-Selectin is not required to grant this protein α-granule localization (Hartwell et al., 1998).

We propose a model in which α-granule proteins must traverse the sorting endosome (Figure 1). MK-synthesized luminal proteins likely concentrate and leave the Golgi in sorting endosome-targeted carriers (Figure 1, step 1) while MK-made transmembrane proteins have the option of reaching the sorting endosome directly or by endocytosis from the plasma membrane (Figure 1, steps 1–4). Similarly, luminal α-granule cargo taken up by megakaryocytes is endocytosed in a receptor dependent or independent way. Several proteins present on the limiting membrane of the α-granule contain the same sorting signals in their cytosolic domains and their extracellular domains interact with integrins, which allows us to speculate they localize to the same plasma membrane patches and are endocytosed in the same transport carriers (Figure 1, steps 3 and 4). To avoid lysosomal degradation, α-granule proteins require a VPS16B/VPS33B-mediated membrane fusion step (Figure 1, step 5) and the retrieval from a degradative fate by the Commander complex (Figure 1, step 6) (Ambrosio et al., 2022; Ambrosio and Di Pietro, 2019). This complex is also responsible for the recycling to the plasma membrane of transmembrane proteins containing a sorting signal in their cytosolic domain that is recognized by SNX17, such as the α-granule proteins integrins β1 and β3, P-Selectin, and the FV and PF4 endocytic receptor LRP-1. We hypothesize NBEAL2 may be instrumental in concentrating both luminal and transmembrane α-granule cargo in Rab4/Rab11 endosomal subdomains redirecting the recycling machinery towards the α-granule biogenesis pathway (Figure 1, steps 7–9) instead of the conventional route to the cell surface. In this way α-granule proteins reach MVBs (Figure 1, step 9). We theorize MVBIIs do not necessarily originate from conventional MVBs. They contain dense proteinaceous material and intra-luminal vesicles (ILVs) decorated with CD63, P-Selectin and αIIbβ3 that resemble more ILVs found in WPBs than conventional MVBs destined for lysosomal degradation (Heijnen et al., 1998; Streetley et al., 2019; Heijnen, 2019). Finally, these MVBIIs mature into α-granules (Figure 1, step 10).

Learning about the biogenesis of α-granules not only has an impact on the understanding of hematopoietic and cardiovascular diseases but also on the ability to manipulate the content of platelet α-granules with potential clinical applications. Great progress has been made in the generation of MKs and platelets from induced pluripotent stem cells (iPSCs) (Nakamura et al., 2014; Ito et al., 2018; Borger et al., 2016; Feng et al., 2014; Liu et al., 2015). Recently, a patient suffering from alloimmune platelet transfusion refractoriness was successfully transfused with tailored platelets generated using her own iPSCs (Sugimoto et al., 2022). Excitingly, it has been shown that the content of *in vitro* grown MK α-granules can be modified by adding proteins to the culture medium that are then endocytosed by the MKs (Zhang and Newman, 2019; Poncz et al., 2024). Moreover, platelets derived from these MKs effectively release these ectopic proteins, opening the door to the use of designer platelets as a new method for the delivery of therapeutics in human disease (Poncz et al., 2024).
